# Iron deficiency causes a shift in AMP-activated protein kinase (AMPK) subunit composition in rat skeletal muscle

**DOI:** 10.1186/1743-7075-9-104

**Published:** 2012-11-21

**Authors:** John F Merrill, David M Thomson, Shalene E Hardman, Squire D Hepworth, Shelby Willie, Chad R Hancock

**Affiliations:** 1 Department of Physiology and Developmental Biology, Brigham Young University, Provo, Utah, USA; 2 Department of Nutrition, Dietetics, and Food Science, Brigham Young University, Provo, Utah, USA

**Keywords:** AMPK, AMPK alpha, Iron deficiency, Anemia, Energy metabolism, Skeletal muscle

## Abstract

**Background:**

As a cellular energy sensor, the 5’AMP-activated protein kinase (AMPK) is activated in response to energy stresses such as hypoxia and muscle contraction. To determine effects of iron deficiency on AMPK activation and signaling, as well as the AMPK subunit composition in skeletal muscle, rats were fed a control (C=50-58 mg/kg Fe) or iron deficient (ID=2-6 mg/kg Fe) diet for 6–8 wks.

**Results:**

Their respective hematocrits were 47.5% ± 1.0 and 16.5% ± 0.6. Iron deficiency resulted in 28.3% greater muscle fatigue (p<0.01) in response to 10 min of stimulation (1 twitch/sec) and was associated with a greater reduction in phosphocreatine (C: Resting 24.1 ± 0.9 μmol/g, Stim 13.1 ± 1.5 μmol/g; ID: Resting 22.7 ± 1.0 μmol/g, Stim 3.2 ± 0.7 μmol/g; p<0.01) and ATP levels (C: Resting 5.89 ± 0.48 μmol/g, Stim 6.03 ± 0.35 μmol/g; ID: Resting 5.51 ± 0.20 μmol/g, Stim 4.19 ± 0.47 μmol/g; p<0.05). AMPK activation increased with stimulation in muscles of C and ID animals. A reduction in Cytochrome c and other iron-dependent mitochondrial proteins was observed in ID animals (p<0.01). The AMPK catalytic subunit (α) was examined because both isoforms are known to play different roles in responding to energy challenges. In ID animals, AMPKα2 subunit protein content was reduced to 71.6% of C (p<0.05), however this did not result in a significant difference in resting AMPKα2 activity. AMPKα1 protein was unchanged, however an overall increase in AMPKα1 activity was observed (C: 0.91 pmol/mg/min; ID: 1.63 pmol/mg/min; p<0.05). Resting phospho Acetyl CoA Carboxylase (pACC) was unchanged. In addition, we observed significant reductions in the β2 and γ3 subunits of AMPK in response to iron deficiency.

**Conclusions:**

This study indicates that chronic iron deficiency causes a shift in the expression of AMPKα, β, and γ subunit composition. Iron deficiency also causes chronic activation of AMPK as well as an increase in AMPKα1 activity in exercised skeletal muscle.

## Background

Iron is important for oxygen transport and ATP synthesis. If these processes are impaired by iron deficiency, cellular adaptations occur, such as an increased glucose dependence, in response to that deficiency [1,2]. The 5’AMP-activated protein kinase (AMPK) has been characterized as a major cellular energy sensor [3], which may mediate some of these adaptations. AMPK is activated in response to energy challenges such as hypoxia, muscle contraction, and hypoglycemia. Therefore, because AMPK is central to how cells respond to changes in the energy status of the cell and iron homeostasis is critical for the transduction of energy within the cell, we set out to investigate the effects of iron deficiency on AMPK activation and signaling. Ultimately we believe that this information may help elucidate why specific cellular responses occur with changes in cellular iron status.

Iron deficiency is the most common worldwide nutrient deficiency. Of the world’s total population, 24.8% of individuals are anemic [4]. Anemia occurs at all stages of the life cycle, in both developing and developed countries, being most prevalent in pregnant women and young children (41.8% and 47.4% respectively) [4]. Iron deficiency is a metabolic stress because it compromises both the capacity for oxygen supply to tissues (anemia), as well as the capacity to utilize oxygen due to impairment of mitochondrial capacity [5]. This type of energetic stress, due to either decreased oxygen supply or utilization, has been shown to cause an increase in AMPK activation [6].

AMPK is a cellular energy sensor that when activated, stimulates catabolic processes that increase ATP synthesis, and concurrently inhibits anabolic processes that consume ATP [7]. Nutritional or environmental stress, such as hypoglycemia, hypoxia, and/or muscle contraction, lead to an increase in the AMP:ATP ratio [8]. The function of the enzyme is altered by the interaction of the AMPK subunits as conformational changes occur. AMPK is a heterotrimer consisting of one alpha catalytic subunit and two regulatory subunits, beta and gamma [9], and multiple isoforms of all subunits have been identified (α1, α2, β1 ,β2, γ1, γ2, γ3) [10,11]. AMPK complexes containing the α2 isoform are more sensitive to changes in AMP concentration than are complexes containing α1 [12]. Furthermore each isoform of the α subunit affects different downstream signaling pathways. For example, the α1 subunit is more important in the inhibition of protein synthesis via the mTOR pathway [13,14]. The activation of AMPK is largely determined by phosphorylation of Thr^172^ on the α subunit, which causes a greater than 20-fold increase in activity [3]. This is primarily done by the predominant AMPK kinase in skeletal muscle, LKB1 [15,16], and is enhanced when the AMP:ATP ratio is high by nucleotide binding to the γ subunit of AMPK [17]. Binding of AMP to the γ subunit increases activation of AMPK by up to fivefold, but also makes AMPK a poorer substrate for the phosphatase and increases phosphorylation by LKB1 (a net increase in activity of >1000-fold) [18,19]. Conversely, when the AMP:ATP ratio is low, the nucleotide binding sites on γ are occupied by ATP, eliminating inhibition of the phosphatase, decreasing net enzymatic activity [20].

Exercise is a metabolic stress on the cell, which has been shown to increase the activation of AMPK [21,22]. Muscle contraction during exercise increases the AMP:ATP ratio because excitation-contraction coupling depends on the hydrolysis of ATP to ADP as its source of energy. Myokinase catalyzes a reaction that transfers a phoshoryl group from one ADP to another, which regenerates one ATP, and forms one AMP (2 ADP → ATP + AMP). This reaction is important for limiting the increase in ADP when the rate of ATP hydrolysis is high, and thus results in an increase in the AMP:ATP ratio [3,23,24]. The concerted effects of iron deficiency and muscle contraction on AMPK activation however are still unknown. A muscle cell that is metabolically stressed due to iron deficiency may be even more adversely affected by exercise than an iron sufficient cell. This increased stress likely has consequences on AMPK activation and signaling.

The effects of hypoxia on AMPK activation have been documented [25], as well as the adverse effects of iron deficiency on mitochondrial enzyme content [5,26,27], and AMPK phosphorylation due to iron deficiency [6]. The objective of this study was to examine: 1) the extent to which chronic AMPK activation occurs due to iron deficiency, 2) how AMPK activation and signaling due to muscle stimulation is affected during iron deficiency, and 3) the effects of iron deficiency on the AMPK subunit composition in skeletal muscle.

## Results

### Degree of iron deficiency

**Figure 1:**
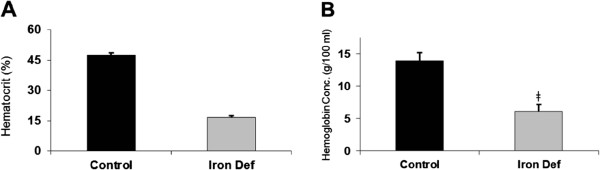
**Plasma hematocrit and hemoglobin concentration. A**. Hematocrit decreased dramatically with iron deficiency (ǂ indicates statistical significance, p<0.01) (n=4). **B**. Hemoglobin concentration decreased significantly with iron deficiency (ǂ indicates statistical significance, p<0.01) (n=4).

Animals fed a diet deficient in iron content became anemic by 6 weeks of feeding. This was evident by a significant reduction of both hematocrit and hemoglobin (Figures 1A and 1B).

**Figure 2:**
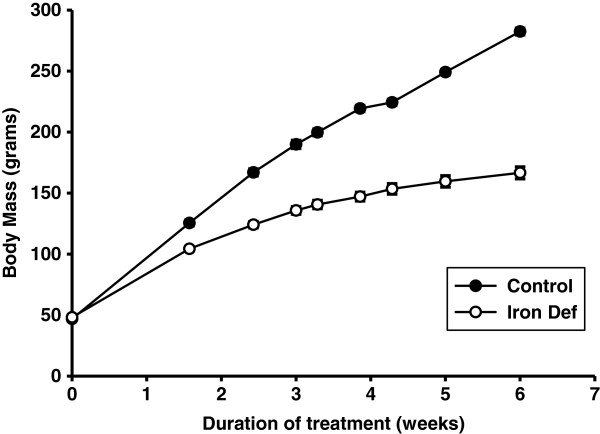
**Mean body mass of control and iron deficient groups.** The rate of growth of the ID group was beginning to decrease by the end of the treatment period, whereas the C group did not show a decrease in growth rate. The mean body mass of the C group was significantly greater than the ID group by day 10 of treatment (p<0.01). A difference that continued to increase throughout the treatment period (n=15).

*Body Mass.* Mean body mass of both C and ID groups were not different on day 0 of treatment at 21 days of age. On day 11, the mean body mass of the C group was 20% greater than that of the ID group. Due to this large disparity between the two groups we began pair feeding the C group to the ID group on day 13. The C group continued, however, to grow at a higher rate than the ID group. After 6 weeks of treatment, the majority of that being pair feeding, the mean body mass of the C group was 282 ± 3 g, while that of the ID group was 167 ± 5 g (Figure 2). The ratio of the tibialis anterior muscle mass to the body mass was measured to determine whether growth of the skeletal muscle was proportionate to total body growth. This ratio was not different between the two groups (C: 1.86 mg muscle weight/g body weight ± 0.06; ID: 1.87 mg muscle weight/g body weight ± 0.03).

### Effect of iron deficiency on mitochondrial enzyme expression

**Figure 3:**
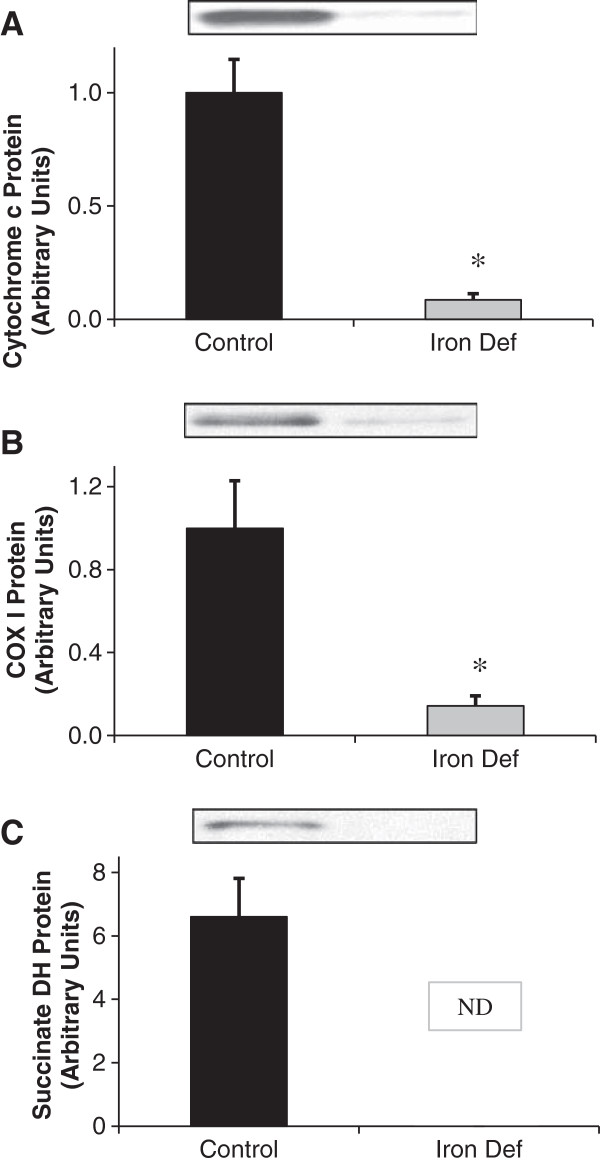
**Abundance of mitochondrial proteins in gastrocnemius muscle.** Iron-containing proteins of the electron transport chain are expected to decrease with iron deficiency. **A**. Cytochrome c protein levels decreased dramatically with iron deficiency (p<0.01). **B**. COX1 protein levels decreased dramatically with iron deficiency (p<0.01). **C**. Succinate dehydrogenase protein levels were undetectable in tissues of ID animals. Since below 2 arbitrary units would have been considered to be significantly different, it can be concluded that the reduction in succinate dehydrogenase was statistically significant.

The electron transport chain (ETC.) of the mitochondria depends on iron as an electron acceptor/donor at multiple steps of electron transport. Cytochrome c, Cytochrome c oxidase I (COX1), and Succinate Dehydrogenase are all iron-dependent enzymes of the ETC. Because they depend on iron for enzymatic activity, it would be expected that their protein content would decrease with iron deficiency. All three of these enzymes decreased significantly with iron deficiency (p<0.01) (Figure 3), confirming that iron deficiency created a situation of significant energy insult.

### Skeletal muscle fatigue

It has been shown that muscle contraction causes an increase in AMPK activity [28]. In order to further show differences in AMPK activation due to iron deficiency, *in situ* muscle stimulation was performed. In past studies with a low-demand stimulation protocol similar to ours, oxygen has been supplied during muscle stimulation, and C rats showed little or no fatigue [29]. Therefore, in the present study, oxygen was supplied to both the C and ID groups to limit the oxygen deficit due to anemia and increase our ability to focus on muscle specific effects of iron deficiency. Evidence that this approach was successful in limiting the hypoxia of the control rats includes: 1) the resting concentration of phosphocreatine was not different between C and ID muscles and 2) a difference in AMPK activity under these conditions was not observed at rest, as others have shown [6]. Although this did serve to limit the oxygen deficit, it did not eliminate it completely. Hemoglobin concentration in the ID rats was reduced by about half, meaning that the ID rats only had about 50% of the oxygen carrying capacity of the C rats. Even with supplementation of 100% oxygen, the ID rats during stimulation were likely hypoxic compared to the C rats.

**Figure 4:**
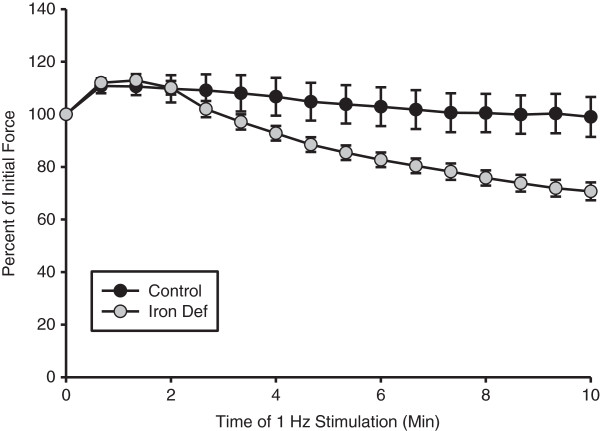
**Force of muscle contraction.** Fatigue was much greater in the ID group as evidence by the drop off in force that they were able to generate after continued 1 Hz muscle stimulation. After 6 min 40 sec the amount of force generated by the C rats was significantly greater than that of the ID rats (p<0.01) (n=11-14).

The mean initial force generated by the C group was 39% greater than that generated by the ID group (C: 601 ± 42 g; ID: 433 ± 34 g). Although the mean body mass of the C group was greater, the amount of force generated by the right hindlimb GPS complex per gram of muscle mass was not different between the two groups (C: 0.24 ± 0.03 g/mg muscle; 0.32 ± 0.04 g/mg muscle). In order to analyze relative fatigue, force data was analyzed as a percent of the initial force generated by each group during the 10 minutes of stimulation. Although within the first 1–2 minutes of stimulation, the amount of force generated by the two groups increased, the force generated by the ID group dropped to 72% of the initial force after 10 minutes of stimulation, whereas the C group was able to generate 100% of the initial force by the end of the same 10-minute period (Figure 4). Thus, this stimulation protocol was mild enough to limit any net fatigue in the control rats over the course of 10 minutes.

### High energy phosphates

Aside from the major mechanisms of ATP synthesis from carbohydrate and fatty acid substrates, the concentration of ATP is buffered through the creatine kinase system. In addition, an increase in the concentration of ADP during exercise is limited by the myokinase reaction (2ADP ←→ ATP + AMP), resulting in an increase in AMP_._ Much of that AMP is then converted to IMP by AMP deaminase. This buffering action by creatine kinase and myokinase as well as the continual supply of ATP from oxidative phosphorylation limits any net reduction in ATP under most energy demands. During high energy demands, muscle AMP and IMP increase, while ATP and phosphocreatine decrease. The purpose of measuring the concentration of high-energy phosphates in these muscles tissues was to determine and compare the cellular energy state of the ID and C groups. An increase in ADP and AMP, is associated with activation of AMPK [30].

Phosphocreatine levels decreased in the C group by 45.9% during the 10-minute stimulation period. In the ID group however, phosphocreatine levels were reduced by 85.9% (Table 1). IMP concentration increased modestly in C rats during stimulation (3.6-fold), but increased by a much larger amount in ID rats (8.7-fold) (Table 1). There was also a large increase in estimated free concentration of AMP with stimulation in the ID rats (2.07 ± 1.06 vs. 0.17 ± 0.09 nmol/g wet wt in ID compared to control muscles), which approached statistical significance (p=0.05). These data confirm that while the stimulation protocol was mild (no net fatigue in the controls) the iron deficient rats sustained a much greater challenge to the energy state.

**Table 1:** High energy phosphate concentrations in gastrocnemius muscle

	**Control**		**Iron Def**		
	**Rest**	**Stim**		**Rest**	**Stim**
ATP (n=6-8)	5.89 ± 0.48	6.03 ± 0.35		5.51 ± 0.20	4.19 ± 0.47ǂ
ADP (n=6-8)	0.79 ± 0.08	0.96 ± 0.07		0.73 ± 0.03	1.03 ± 0.07
AMP (n=6-8)	0.06 ± 0.01	0.09 ± 0.02		0.05 ± 0.00	0.16 ± 0.06
IMP (n=6-8)	0.22 ± 0.01	0.80 ± 0.30		0.26 ± 0.02	2.26 ± 0.63ǂ
PCr (n=9-10)	24.1 ± 0.9	13.1 ± 1.5		22.7 ± 1.0	3.2 ± 0.7ǂ
Cr (n=9-10)	15.8 ± 1.1	28.3 ± 2.3		19.2 ± 0.9	37.0 ± 1.8ǂ

Values are means ± SE in α mol/g wet wt. *PCr*, phosphocreatine; *Cr*, creatine.

ǂ Significantly different from control (p<0.01).

### Analysis of AMPK activation

**Figure 5:**
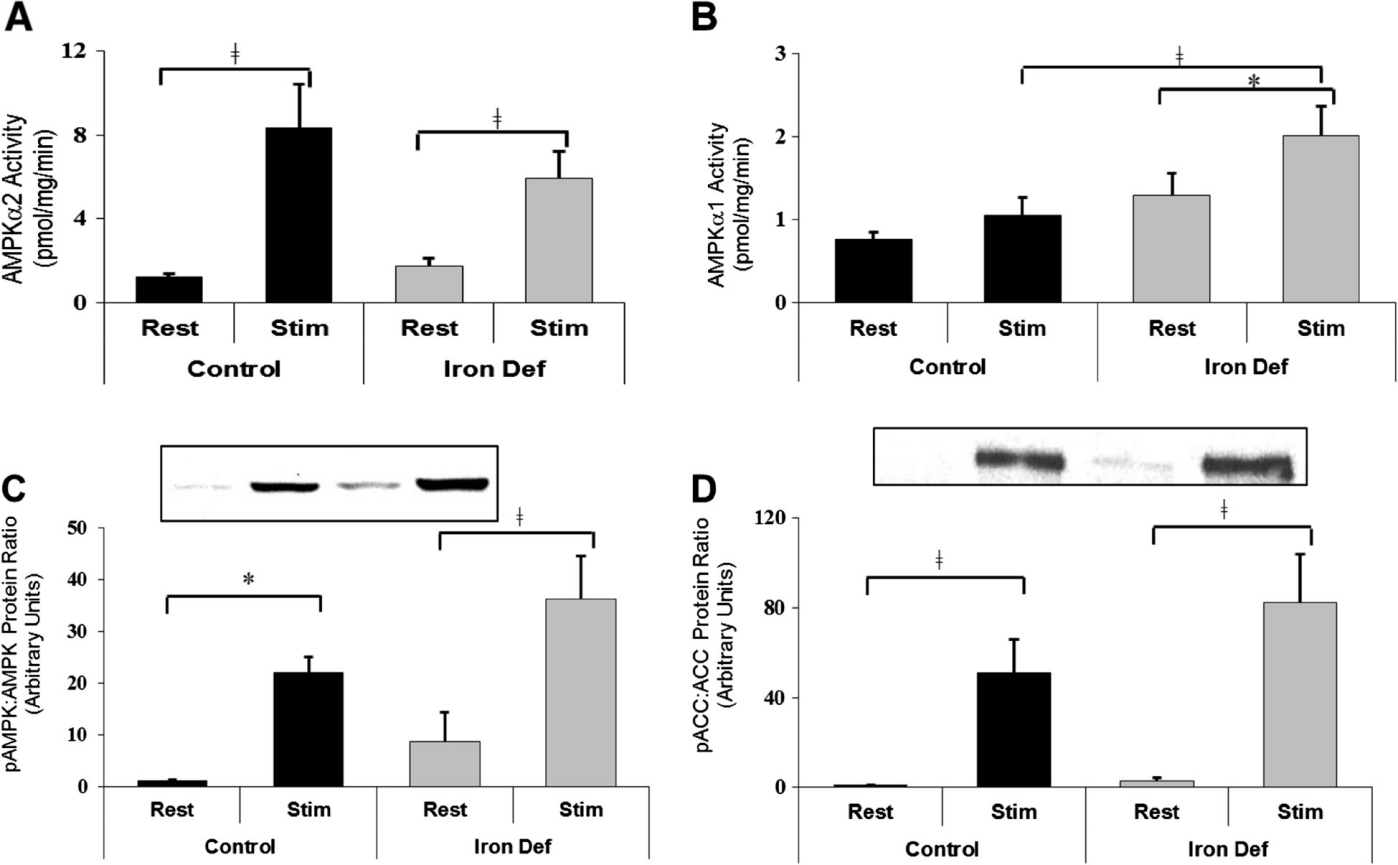
**Acute activation of AMPK in gastrocnemius muscle. A**. AMPKα2 activity in resting limb did not increase with iron deficiency. AMPKα2 increased with stimulation in both C and ID groups (* indicates statistical significance, p<0.05) (n=9-10). **B**. AMPKα1 activity in resting limb did not increase with iron deficiency. AMPKα1 activity in stimulated limb did increase with iron deficiency (ǂ indicates statistical significance, p<0.01). Also, activity of α1 did not increase with muscle stimulation in the C rats, whereas it did increase with stimulation in the ID rats (* indicates statistical significance, p=0.05) (n=8-10). **C**. Total AMPK phosphorylation did not significantly increase with iron deficiency. It did however increase with stimulation in both the C (* indicates statistical significance, p=0.05) and ID groups (ǂ indicates statistical significance, p<0.01) (n=3-4). **D**. Phosphorylation of ACC, a direct target of AMPK, did not significantly increase with iron deficiency, but did increase with stimulation in both C and ID groups (ǂ indicates statistical significance, p<0.01) (n=6-8).

After establishing that the ID rats were iron deficient and that this did create a metabolic stress, we examined the effects of chronic iron deficiency and *in situ* muscle stimulation on activation of the two AMPKα isoforms. In accordance with previous findings [21,22], the overall effect of stimulation was a significant increase in activation of both AMPKα isoforms in both the C and ID groups. The two AMPKα isoforms, however, were affected differently by iron deficiency. Although it has been shown to be more AMP-dependent than AMPKα1 [12], resting AMPKα2 activity was not significantly different between the C and ID groups (Figure 5A). Iron deficiency however did cause an increase in resting AMPKα1 activity which approached statistical significance (p=0.05). Also, activation of AMPKα1 did not increase significantly due to stimulation in the C group alone, but in the ID group, stimulation did result in a significant increase in AMPKα1 activation (p=0.05) (Figure 5B).

Since AMPK is activated by phosphorylation, the ratio of the expression of phosphoAMPK to total AMPKα is also a reflection of total AMPK activation. This ratio did not increase significantly due to iron deficiency. It did, however increase with stimulation of both the C and ID groups (Figure 5C).

Acetyl CoA Carboxylase (ACC) is a downstream substrate of AMPK, and is inhibited by phosphorylation. The ratio of expression phosphoACC to total ACC was therefore used as an additional indicator of AMPK activation. Although ACC phosphorylation did increase greatly with stimulation, it did not increase significantly in rested tissues with iron deficiency (Figure 5D).

### Analysis of AMPK subunit expression

**Figure 6:**
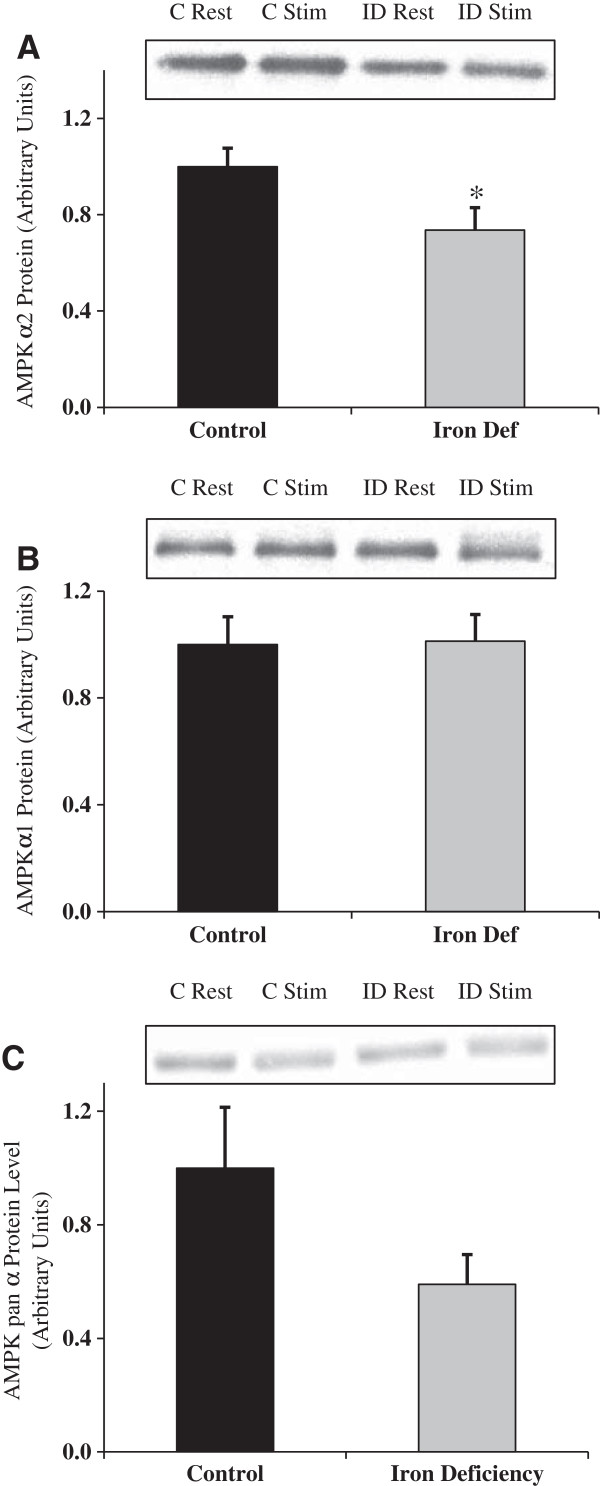
**AMPK alpha subunit expression in gastrocnemius muscle with iron deficiency anemia. A**. AMPKα2 protein content decreased with iron deficiency (* indicates statistical significance, p<0.05) (n=5-6). **B**. AMPKα1 protein content was not different between the two groups (n=8). **C**. Total AMPK pan α protein content did not change with iron deficiency (n=4). The decrease in total AMPKα is not surprising given that α2 is the more abundant isoform found in skeletal muscle. The decrease in α2, with no concurrent decrease in α1 indicates a slight shift in α subunit composition due to iron deficiency.

**Figure 7:**
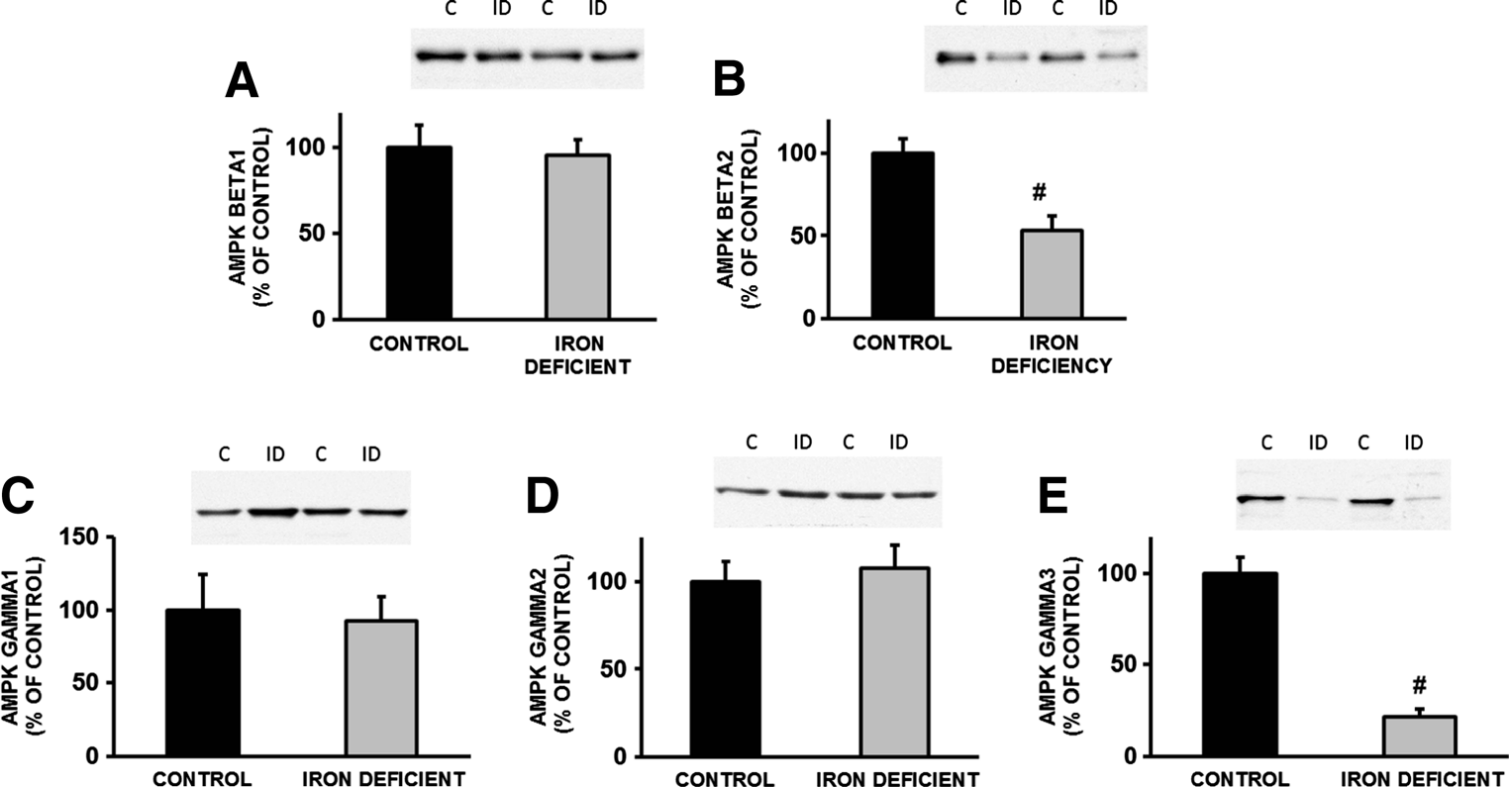
**AMPK beta and gamma subunit expression following irion deficiency anemia in rat gastrocnemius muscle. A**. AMPK b1 subunit expression was not different in response to iron deficiency. **B**. AMPK b2 subunit expression was reduced with iron deficiency in response to iron deficiency (p<0.01). No changes were observed with AMPK g1 (**C**) or g2 (**D**) subunit expression in response to iron deficiency, however a marked reduction was observed with g3 subunit expression (p<0.001) (**E**).

*Alpha subunits:*AMPK complexes containing the α2 isoform sense and respond differently to energy stresses than AMPK complexes containing the α1 isoform. AMPKα2 is more sensitive to changes in AMP concentration than AMPKα1 [12]. AMPKα2 protein content decreased significantly (p<0.05) (Figure 6A), while AMPKα1 did not change significantly (Figure 6B). These data suggest a slight shift in the AMPKα subunit composition due to the chronic stress of iron deficiency. A non-isoform-specific AMPK pan α antibody was also used to quantitate expression of total AMPK, which was determined to be unchanged with iron deficiency (Figure 6C). *Beta and gamma subunits:* In addition to the α subunit expression, we also examined the expression of the regulatory β and γ subunits. While there were no differences in the expression of the β1, γ1 and γ2 subunits (Figure 7A, C and D), there was a significant reduction in both the β2 (Figure 7B) and γ3 (Figure 7E) subunits in response to severe iron deficiency.

### Chronic activation of AMPK

**Figure 8:**
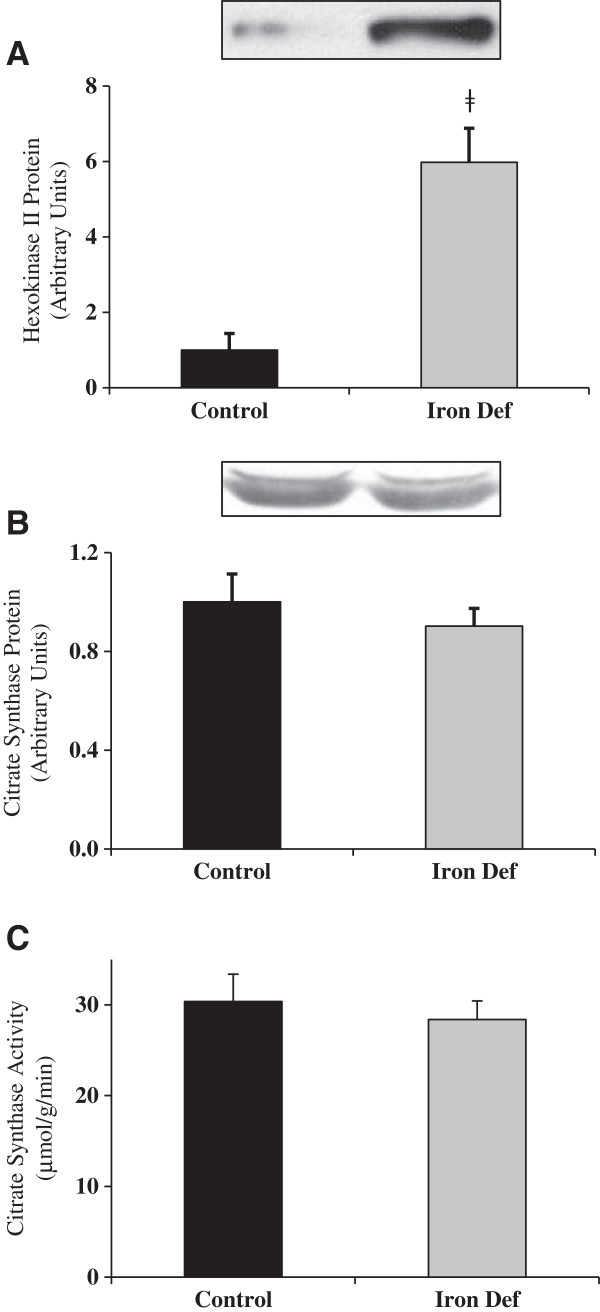
**Severe iron deficiency, hexokinase and citrate synthase expression in gastrocnemius muscle. A**. Hexokinase expression increased significantly with iron deficiency (ǂ indicates statistical significance, p<0.01) (n=6). **B**. There was no difference in citrate synthase expression between C and ID groups (n=6). **C**. There was no difference in citrate synthase activity between C and ID groups (n=7-8).

Treatment with AICAR (a chemical activator of AMPK) has been shown to increase expression of Hexokinase II in skeletal muscle [31]. Therefore, we examined Hexokinase II expression as an indicator of chronic AMPK activation and found it to be increased dramatically with iron deficiency (p<0.01) (Figure 8A).

Citrate synthase is the first enzyme in the citric acid cycle. It can be upregulated by chronic activation of AMPK. Western blot data for citrate synthase revealed that its expression was not changed with iron deficiency (Figure 8B). Citrate synthase activity data also revealed no change with iron deficiency (Figure 8C).

## Discussion

AMPK is a key regulator of cellular energy homeostasis. It becomes activated by various energy challenges which result in an increase in the AMP:ATP ratio. Iron deficiency is one such energy challenge. It results in a decrease in the iron-containing enzymes of the electron transport chain, without affecting the non-iron dependent enzymes of the citric acid cycle [27]. These changes lead to a decrease in muscle respiratory capacity as well as increased susceptibility to fatigue [26]. The ability of AMPK to sense energetic insults such as this leads to the question as to whether its activity increases, and how its expression and signaling are affected during iron deficiency. Following 6–8 weeks of treatment, AMPKα2 expression in ID animals was reduced significantly compared to C (p<0.05). AMPKα1 expression was not different as a result of iron deficiency. Under the conditions in this study, iron deficiency did not result in a difference in resting AMPKα2 activity. Interestingly, iron deficiency did cause an increase in resting AMPKα1 activity, which approached statistical significance (p=0.05). Also, muscle stimulation did not result in increased activation of AMPKα1 in the C group, but in the ID group, stimulation did result in a significant increase in AMPKα1 activity (p=0.05). When the β and γ subunits were examined, significant reductions were observed in both the β2 and γ3 subunits of AMPK. The novel findings of this study are that chronic iron deficiency causes a shift in the expression of AMPKα, β and γ subunit composition as well as potentially altered sensitivity of AMPKα1 and AMPKα2 to energy challenges such as muscle contraction.

### Iron deficiency anemia causes a shift in AMPK subunit composition

Muscle contraction in ID rats resulted in a greater increase in AMPKα1 activation than in C rats. Increased AMPK phosphorylation, or covalent activation of AMPK, occurs when an increase in the AMP:ATP ratio results in AMP binding to the γ subunit. More recent work has also proposed a role for ADP in addition to AMP in the allosteric regulation of AMPK (see recent review by Hardie, Carling and Gamblin [30]). AMP binding directly to the γ subunit results in allosteric activation of AMPK, which alone, increases activity by up to fivefold [18]. AMPKα2 is more responsive to an increase in the concentration of free AMP (AMP_f_) than AMPKα1 [12]. In our calculation of the difference of AMP_f_ concentration between C and ID groups with stimulation, we did not achieve strong statistical significance because of the large variability between samples. An increase in AMP_f_ concentration however, can be assumed based on the significant increase in IMP concentration, since IMP is a product of AMP deamination. Interestingly, we did not see a change in AMPKα2 activity with iron deficiency, even though the HPLC data confirms an increase in the concentration of IMP in the iron deficient, stimulated muscles. Of particular interest is the finding that AMPKα1 activity did increase with iron deficiency. Therefore, there is an apparent inconsistency with the energy status and the AMPK activity data. The protocol used to assess AMPK activity however, fails to reflect the allosteric activation of AMPK that is to be expected with the elevated levels of IMP and AMP_f_ that were seen in the ID rats following muscle stimulation. Unlike our *in vitro* method of measuring AMPK activity, measuring phosphorylation of ACC should account for the allosteric activation of AMPK, as this is a method of measuring total *in vivo* AMPK activity. However, protein analyses indicate that muscle stimulation in the ID rats did not cause a greater increase in ACC phosphorylation than in C rats. This suggests that the reasons for the increase in AMPKα1 activity with muscle stimulation in the ID group may be due to more than just the change in energy status. One possible explanation is the fact that AMPKα1 activity has been shown to increase with overload of the plantaris muscle in LKB1^−/−^ mice, with a concurrent increase in CaMKK activity [32]. This suggests that AMPKα1 is the preferred target for CaMKK, a known upstream kinase of AMPK. Further testing to determine if CaMKK activation is altered due to iron deficiency is necessary in order to confirm this assumption.

Since activation of AMPKα1 increased significantly with stimulation in ID animals, we conclude that there may be an increase in the contribution of AMPKα1 to total AMPK activity with iron deficiency. This is not to say that the contribution of AMPKα1 became greater than that of AMPKα2 to total AMPK activity (AMPKα2 activity still increased more with stimulation in the ID group than AMPKα1 activity). AMPKα1 simply became more responsive to stimulation in the ID group than it was in the C group. This increase in AMPKα1 contribution to total AMPK activity is further confirmed by the finding that AMPKα2 expression decreased with iron deficiency, whereas AMPKα1 expression did not change. Together, these data indicate that iron deficiency may cause a shift toward AMPKα1 having a greater role in the activity of AMPK in skeletal muscle.

In addition to the alpha subunit composition, the composition of the β and γ also determine how AMPK responds to energy stress as well as what downstream effectors are regulated. The heterotrimeric composition that is primarily activated in skeletal muscle is the α2/β2/γ3 combination [33-35]. We report that along with a reduction in the α2 subunit isoform, there is also a marked reduction in the α2 and γ3 subunit isoforms in response to iron deficiency. Previous reports looking at changes in the heterotrimeric subunit composition of AMPK have been somewhat mixed. In response to endurance exercise training in rats, one group has reported fast twitch muscles have an increase in the γ3 subunit protein content with no change in the α and β subunits [36]. Chronic low-frequency stimulation induces an increase in both α subunits [37]. In human muscle, exercise training has been shown to cause an increase in α1, β2 and γ1 subunits and cause a reduction in the γ3 subunit [38,39]. Interestingly, recent work examining the role of γ2 subunit of AMPK suggests that this subunit is critical for AMPK to respond to changes in energy state [40]. Thus, given the reduction observed in the β2 subunit with iron deficiency, we might have expected a blunted response to the energy demands of muscle contractions; however we did not observe this. As noted above, the regulatory effect of AMP on AMPK occurs via binding to the γ subunit. The γ3 isoform has the lowest AMP dependence and the γ1 isoform has the greatest AMP dependence [35]. This shift in the gamma subunit isoform expression that we observed (lower γ3 while maintaining γ1) may contribute to the increase in the sensitivity to muscle contractions observed with α1 activity. Overall, the shift in the protein expression of the AMPK subunit composition did not result in a dramatic reduction or increase in AMPK activity at rest or in response to muscle contractions.

### Evidence of chronic AMPK activation

Han et al. showed that iron deficiency causes chronic activation of AMPK [6]. This is not surprising since iron deficiency causes a decrease in the oxygen carrying capacity of blood as well as a decrease in mitochondrial capacity. The stress that this puts on energy metabolism should be enough to cause chronic AMPK activation due to the mild energy demands of normal ambulatory activity. As reflected by the phosphorylation state of AMPK and ACC however, we did not measure such an increase in chronic AMPK activity. This was likely due to the supplementation of the anesthetized rats with pure oxygen (~40-50 minutes prior to dissection of rested muscles). Based on previous work, this is enough time for any elevation in AMPK activity at rest to return to more normal values [41]. Therefore, due to the length of time during which rested animals were provided with pure oxygen, the effects of iron deficiency on resting muscle AMPK activity were likely masked.

Chronic AMPK activation increases expression of certain mitochondrial and glycolytic enzymes [31,42]. Hexokinase II is regulated by CREB, which can also be phosphorylated by AMPK [43,44]. In support of chronic AMPK activation, hexokinase II expression did increase with iron deficiency. In the present study, iron deficiency presents a unique energy challenge in that many oxidative enzymes of the electron transport chain, enzymes that are generally increased by AMPK activation, are iron-dependent, however all iron-dependent mitochondrial proteins examined were severely reduced due to iron deficiency. Citrate synthase, a non-iron dependent mitochondrial enzyme, was not reduced with iron deficiency. Two possible explanations for this result are: 1) AMPK was not chronically activated with iron deficiency, or 2) iron deficiency has an overall negative impact on mitochondrial content, including transcription of all mitochondrial proteins, and chronic AMPK activation actually prevents down-regulation of the non-iron dependent enzymes, rescuing the cell from complete mitochondrial deficiency. Due to the known effect of AMPK activation on citrate synthase, further work is needed to determine if AMPK is playing a role in preserving citrate synthase expression.

A decrease in important mitochondrial enzymes, as seen in the present study, reflects a significant decrease in muscle oxidative capacity, and is therefore a possible explanation for the stunted growth seen in the ID rats. Skeletal muscles depend on oxidative phosphorylation far more than any other process for the conversion of nutrients to usable forms of energy. In this case it would be expected that any ingested nutrients would not be efficiently used for the production of ATP necessary for protein synthesis and normal growth.

Previous studies have shown that iron deficiency leads to in increased dependence on glucose metabolism [1,2]. The decrease in mitochondrial proteins seen with iron deficiency in previous [27], as well as in the present study, support these findings that iron deficiency causes a shift away from oxidative metabolism. A novel finding shown here is a significant increase in Hexokinase II expression, supporting previous findings that iron deficiency causes a shift toward glycolytic metabolism in order to compensate for the loss of oxidative capacity. As previously stated, chronic AMPK activation increases hexokinase II expression, supporting the idea that AMPK is at least in part responsible for the shift toward glycolytic metabolism seen with iron deficiency. A transgenic model in which AMPK activation is prevented might provide insight into this question.

Another possible explanation for the stunted growth seen in the ID rats is the role of AMPK in inhibition of the actions of mTOR. As previously stated, AMPKα1 is more important than AMPKα2 in the inhibition of protein synthesis via the mTOR pathway [14,32]. This shift toward AMPKα1 having a greater role in total AMPKα activity is another possible explanation for the large difference in body mass between the C and ID groups. If AMPKα1 proportionally increases with iron deficiency, the mTOR pathway would be further inhibited in ID animals than in C animals, causing increased inhibition of protein synthesis, and therefore decreased growth.

## Conclusions

As a cellular sensor and regulator of energy homeostasis, AMPK senses and responds to many energy challenges, such as iron deficiency, in a variety of ways. Here we provide evidence for a chronic increase in AMPK activity. We also show a decrease in AMPKα2, β2 and γ3 AMPK subunits, with no concurrent change in AMPKα1, β1, γ1 or γ2 proteins, resulting in an overall shift in AMPK subunit composition. These data suggest that iron deficiency results in a possible shift toward an increase in the role of AMPKα1/ β1/γ1 heterotrimer dependent signaling in total AMPK regulation of cellular energy homeostasis. Iron deficiency also causes a shift away from oxidative and toward glycolytic metabolism in rat skeletal muscle. Taken together, these results lead to the question as to whether AMPK is responsible for the metabolic shift seen with iron deficiency. Future work needs to be done in order to determine if such is the case.

## Methods

### Animals

Male Wistar rats were kept in a temperature controlled and well-ventilated room with a 12:12 hour light dark cycle. They were kept in stainless steel mesh wire bottom cages, with no bedding material, and no access to feces. All rats were given free access to distilled water. The iron deficient (ID) group was fed *ad libitum* and the control (C) group was pair fed to the ID group (see below). All experimental procedures were approved by the institutional animal care and use committee of Brigham Young University.

#### Body weight

The rats were weighed 2–3 times per week from the first day on the diets up to, and including the day of sacrifice.

### Treatments

The ID rats were fed an iron deficient diet *ad libitum* for 6–8 weeks (n=15). The iron deficient diet was obtained from Teklad Lab Animal Diets (Harlan Laboratories, Madison, WI), and consisted of: Casein (low Cu & Fe), DL-methionine, sucrose, corn starch, corn oil, mineral mix (Fe deficient), vitamin mix, choline bitartrate, ethoxyquin) (TD.80396, contains approximately 2–6 ppm Fe). The C rats were fed the same diet with 48 ppm added iron (TD.80394), pair fed to the ID group for the same period of time (n=15). All rats were approximately 21 days of age on day 0 of treatment.

*In situ* muscle stimulation was performed as described previously [24]. Briefly, the rat was anesthetized with 60 mg/kg sodium pentobarbital ip and the sciatic nerve was isolated and stimulated at rate of 1 twitch contraction per second for 10 minuntes (0.25 ms square wave, 6–7 V, using Grass S88X stimulator). In order to limit the oxygen deficit due to reduced hemoglobin in the blood, the anesthetized rat of both C and ID groups was provided with 100% oxygen throughout the procedure.

### Tissue analysis

#### Dissections

After 6–8 weeks of treatment and right hind-limb stimulation, gastrocnemius, soleus, plantaris, and mixed quadriceps muscles were removed quickly and clamp frozen with liquid nitrogen chilled metal tongs and stored at −90°C. The stimulated right hind limb muscles were removed and frozen prior to the rested left hind limb muscles. Whole blood was also collected to determine hemoglobin concentration and percent hematocrit (described below).

#### Muscle contractions

In order to examine the response of AMPK to an increase in energy demands with and without iron deficiency anemia we performed a mild in situ muscle stimulation protocol. Muscle contractions were elicited using the stimulation procedure via direct stimulation of the sciatic nerve as described previously [24]. This stimulation protocol is sufficient to activate all of the muscle fibers that are innervated by the sciatic nerve. The fatigue profile of the gastrocnemius-plantaris-soleus (GPS) complex of muscles was determined in response to a mild contraction protocol consisting of direct sciatic nerve stimulation at a rate of 1 pulse per second. Muscle force was measured using a force transducer (Grass FT103) and performance data was collected using data acquisition software (iWORX). Following the muscle stimulations, the aforementioned calf muscles were quickly dissected from the right hind-limb and flash-frozen with tongs maintained in liquid nitrogen. Corresponding muscles in the contra-lateral unstimulated limb were subsequently dissected and frozen as rested control muscles.

#### Homogenization

Frozen gastrocnemius muscles were pulverized in liquid nitrogen, weighed, and homogenized as a 5% solution in homogenization buffer (20 mM Tris–HCl, 250 mM Mannitol, 50 mM NaF, 5 mM Sodium Pyrophosphate, 1 mM EDTA, 1 mM EGTA, 1% Triton X-100, 50 mM β-glycerophosphate, 1 mM Sodium Orthovanadate, 1 mM DTT, 1 mM Benzamadine, 0.1 mM PMSF, 5 βg/ml Soybean Trypsin Inhibitor, pH 7.4) (50 mg muscle powder/1 ml buffer) then stored in −90°C freezer.

#### Packed cell volume (hematocrit)

Blood was taken from the tail 4 weeks after the start of feeding, and once weekly after that, to determine degree of iron deficiency anemia. This was done by drawing blood into a capillary tube and centrifuging for 5 min at 14,500 rpm.

#### Protein quantification

The standard Lowry Protein Assay protocol (BioRad, Hercules, CA) was used to measure the protein concentration in each muscle homogenate so that an equal amount of protein was added to each well for electrophoresis.

#### Hemoglobin quantification

A hemoglobin quantification assay was used to determine the degree of anemia reached in the ID rats versus the C rats on the day of sacrifice as previously described [45,46]. Briefly, known concentration hemoglobin standards were made by adding the appropriate amount of hemoglobin to the appropriate volume of Drabkin’s solution (1 vial of Drabkin’s reagent with 1000 ml water and 0.5 ml Brij 35 Solution). Abs_540nm_ was recorded and used to make a linear standard curve. Whole blood samples were diluted 10-fold in 0.9% NaCl solution. 2.5 ml Drabkin’s solution was added to a series of test tubes. 10 μl of whole blood sample was added to each test tube and allowed to sit for 15 min at room temperature. Abs_540nm_ was recorded and compared with standard curve to determine hemoglobin concentration of each sample.

#### AMPK activity assay

An AMPK Activity Assay was used to directly measure and compare the amount of AMPK that was activated in the different experimental groups. The protocol used was described previously [21,47]. Briefly, immunoprecipitation was performed on spun muscle homogenates in order to isolate AMPKα1 and AMPKα2 with the appropriate antibodies (AMPKα1 1:10,000 from Bethyl – cat. no. A300-507A; AMPKα2 1:4,000 from Bethyl – cat. no. A300-508A). AMPK activity was quantitated by measuring the rate of incorporation of radiolabeled phosphate from ATP into an artificial peptide substrate (SAMS) with a sequence similar to that of liver acetyl-CoA carboxylase, a natural substrate for AMPK.

#### Citrate synthase activity assay

A citrate synthase assay was used to measure the difference in activity of the enzyme between the C group and the ID group as previously described by Srere [48].

#### Western Blot

Standard Western blot protocol was used. Western blot analysis was performed on gastrocnemius muscles for AMPKα1 (1:10,000 from Bethyl; cat. no. A300-507A), AMPKα2 (1:4,000 from Bethyl; cat. no. A300-508A), AMPKβ1(1:4,000 from Cell Signaling; cat. no. 4182), AMPKβ2 (1:4,000 from Cell Signaling; cat. no. 4148), AMPKγ1 (1:4,000 from Cell Signaling; cat. no. 4187), AMPKγ2 (1:2,000 from Santa Cruz; cat. no. 20165), AMPKγ3 (1:2,000 custom made from Affinity Bioreagents), AMPK pan α (1:2,000 from Cell Signaling; cat. no. 2532L), phosphoAMPKα (1:1,000 from Cell Signaling; cat. no. 4188L), ACC (1:2,000 from Cell Signaling; cat. no. 3662), phosphoACC (1:5,000 from Cell Signaling; cat. no. 3661S), hexokinase II (1:10,000 from Santa Cruz; cat. no. sc-6521), myoglobin (1:3,000 from Santa Cruz; cat. no. sc-25607), cytochrome c oxidase complex 1 (COX-1) and succinate dehydrogenase (1,5:000 OxPhos Cocktail Ab from Invitrogen; cat. no. 458009), and cytochrome c (1:5,000 from Sigma; cat. no. C5723). Western blot analysis was also performed on mixed quadriceps muscle for citrate synthase (1:10,000 from Alpha Diagnostic; cat. no. CISY11-A).

#### High Performance Liquid Chromatography (HPLC)

Preparation of tissues for HPLC were done using the Perchlorate Tissue Extraction method as described by Chen [49]. Briefly, muscles were ground to powder at liquid nitrogen temperature and then homogenized in 3.5% perchloric acid in a volume 19 times the wet weight of the muscle. The homogenates were then centrifuged at 12,000 rpm for 7 min to remove protein. This was followed by neutralization of the supernatant with tri-n-octylamine and 1,1,2-trichlorotrifluoroethane. After vortexing for 1 min, the neutralized homogenates were again centrifuged at 2,000 rpm for 7 min. The top phase was saved, checked for pH (it should be between 5.5-6.5), and stored at −80°C. Adenine nucleotides (ATP, ADP, AMP) and IMP were quantified by reverse-phase HPLC, as described by Tullson et al. [50]. Phospocreatine (PCr) and creatine (Cr) concentrations were measured by ion exchange HPLC as described by Wiseman and colleagues [51].

Free ADP ([ADP_f_) and free AMP concentrations ([AMP_f_) were estimated based on the following equations

$$\left[{\text{ADP}}_{\text{f}}\right]\;=\;\left[\text{ATP}\right]\left[\text{creatine}\right]/K_{\text{obs}}\left[{\text{H}}^{+}\right]\left[\text{phosphocreatine}\right]$$

where *K*_obs_ = 1.66 × 10^-6^ mM^-1^, and assuming 76% water, and 14% vascular volume [52]

$$\left[{\text{AMP}}_{\text{f}}\right]\;=\;{\left[{\text{ADP}}_{\text{f}}\right]}^{2}/K_{\text{obs}}\left[\text{ATP}\right]$$

where *K*_obs_ = 1.05 × 10 [53]. pH was assumed to be 7 in rested animals [53] however based on the differences in phosphocreatine, fatigue, and IMP accumulation, we estimated pH to be 6.6 in the C animals following stimulation and 6.2 in the ID animals following stimulation [53].

### Statistics

For comparisons including iron deficiency and muscle stimulation (4 different groups) two-way analysis of variance was used. Fisher’s least significant difference post-hoc test was applied where appropriate. When making a simple comparison between ID and C a student’s t-test was used. SigmaStat statistical software was used. Statistical significance is defined as p<0.05. Results are presented as means ± SEM.

## Competing interests

The authors declare that there are no competing interests.

## Authors’ contributions

CRH and JFM, conceived of the study and participated in its design, data collection, analysis and writing of the manuscript. DMT participated in the study design and writing of the manuscript. SDH and SW collected data and analyzed results. All authors read and approved the final manuscript.
